# Digital Epidemiology of Prescription Drug References on X (Formerly Twitter): Neural Network Topic Modeling and Sentiment Analysis

**DOI:** 10.2196/57885

**Published:** 2024-08-23

**Authors:** Varun K Rao, Danny Valdez, Rasika Muralidharan, Jon Agley, Kate S Eddens, Aravind Dendukuri, Vandana Panth, Maria A Parker

**Affiliations:** 1 Department of Epidemiology & Biostatistics School of Public Health Bloomington Indiana University Bloomington Bloomington, IN United States; 2 Department of Applied Health Science School of Public Health Bloomington Indiana University Bloomington Bloomington, IN United States; 3 Luddy School of Informatics, Computing and Engineering Indiana University Bloomington Bloomington, IN United States

**Keywords:** digital epidemiology, BERTtopic, Valence Aware Dictionary and Sentiment Reasoner, VADER, sentiment analysis, social media, prescription drugs, prescription, prescriptions, drug, drugs, drug use, platform X, Twitter, tweet, tweets, latent Dirichlet allocation, machine-driven, natural language processing, NLP, brand name, logistic regression, machine learning, health informatics

## Abstract

**Background:**

Data from the social media platform X (formerly Twitter) can provide insights into the types of language that are used when discussing drug use. In past research using latent Dirichlet allocation (LDA), we found that tweets containing “street names” of prescription drugs were difficult to classify due to the similarity to other colloquialisms and lack of clarity over how the terms were used. Conversely, “brand name” references were more amenable to machine-driven categorization.

**Objective:**

This study sought to use next-generation techniques (beyond LDA) from natural language processing to reprocess X data and automatically cluster groups of tweets into topics to differentiate between street- and brand-name data sets. We also aimed to analyze the differences in emotional valence between the 2 data sets to study the relationship between engagement on social media and sentiment.

**Methods:**

We used the Twitter application programming interface to collect tweets that contained the street and brand name of a prescription drug within the tweet. Using BERTopic in combination with Uniform Manifold Approximation and Projection and k-means, we generated topics for the street-name corpus (n=170,618) and brand-name corpus (n=245,145). Valence Aware Dictionary and Sentiment Reasoner (VADER) scores were used to classify whether tweets within the topics had positive, negative, or neutral sentiments. Two different logistic regression classifiers were used to predict the sentiment label within each corpus. The first model used a tweet’s engagement metrics and topic ID to predict the label, while the second model used those features in addition to the top 5000 tweets with the largest term-frequency–inverse document frequency score.

**Results:**

Using BERTopic, we identified 40 topics for the street-name data set and 5 topics for the brand-name data set, which we generalized into 8 and 5 topics of discussion, respectively. Four of the general themes of discussion in the brand-name corpus referenced drug use, while 2 themes of discussion in the street-name corpus referenced drug use. From the VADER scores, we found that both corpora were inclined toward positive sentiment. Adding the vectorized tweet text increased the accuracy of our models by around 40% compared with the models that did not incorporate the tweet text in both corpora.

**Conclusions:**

BERTopic was able to classify tweets well. As with LDA, the discussion using brand names was more similar between tweets than the discussion using street names. VADER scores could only be logically applied to the brand-name corpus because of the high prevalence of non–drug-related topics in the street-name data. Brand-name tweets either discussed drugs positively or negatively, with few posts having a neutral emotionality. From our machine learning models, engagement alone was not enough to predict the sentiment label; the added context from the tweets was needed to understand the emotionality of a tweet.

## Introduction

### Current State of Social Media for Public Health Surveillance

Social networking websites such as X (formerly Twitter), Facebook, and Instagram are often described as “digital town squares” [1], where people can openly and freely have conversations and discussions about nearly any topic or issue, including those that may not be legal, ethical, or socially acceptable. The broad use and open nature of these conversations have led researchers to use social media to monitor and surveil real-world issues pertaining to public health [2-5]. For example, previous studies have analyzed social media data to develop a real-time influenza surveillance dashboard [6]; monitor the language associated with stress, loneliness, and anxiety during the early months of the US COVID-19 outbreak [7,8]; and track public responses to critical news cycles [9], including recent shifts in US abortion legality [10]. These types of projects focus on extrapolating “real-world” data (such as prevalence rates of influenza or anxiety) from social media discourse. Importantly, numerous practical, analytic, and ethical issues remain to be studied and addressed around the use of social media data for projects that have the potential to directly or immediately impact public or personal health [3,11].

There is a subtle distinction between work seeking to estimate health-related factors such as disease prevalence rates from social media (ie, as described in the previous paragraph) and research specifically focused on understanding public conversations and discourse on social media. There are likely still biases inherent in such analyses (eg, nonindependence of data, platforms’ algorithmic drivers of conversation, and trending topics) [3]. At the same time, analysis of discourse does not attempt to extrapolate secondary or tertiary data points outside of the dialogue itself. Instead, it approaches social media as a lens through which we can view naturally occurring conversations to provide insights about the “state of discourse” in the population of social media users. Such conversations have been studied around a diverse multitude of topics, such as national parks in South Africa [12], healthy diets [13], COVID-19 vaccines [5], and mental health during Mental Health Awareness Week [14].

### Whose Conversations and Discourse Can Readily Be Studied?

A substantial majority of US persons aged 18 to 64 years use social media, as do nearly half of those aged ≥65 years [15]. Therefore, large-scale analyses of posts on social media can be used to infer how the general population might feel about specific issues (though with caution, as noted in the previous paragraph [3]). One important caveat, though, is that different platforms have different user demographics, and people use different platforms at various rates [16]. For instance, data from several years ago suggest that users of Facebook tend to be older (aged ≥50 years), while users of X (Twitter) tend to be younger (aged 18-29 years) [16,17]. These social media dynamics, which may change over time, can influence the types of content that users post and view.

According to a Pew survey on teenage social media use [18] in 2022, about 54% of all teens reported that it would be difficult to give up social media, and among teens who view social media use positively, 46% of teens reported that the main reason they use social media is to connect and socialize with others [19]. Previously, we noted that X (Twitter) is primarily used by younger populations. On X (Twitter), individuals can not only connect with one another but can also become part of web-based communities that discuss diverse topics.

### Learning About Drug Use From Social Media Discourse

The United States is in the midst of a drug overdose epidemic that, in recent years, has claimed >100,000 lives every 12 months [20]. While major strides have been made in attenuating the harm from this crisis, such as increasing access to naloxone [21,22] and harm-reduction strategies [23], the persistence of harmful outcomes associated with drug use suggests that additional strategies and information are needed.

Typically, information about drug use is obtained from investigator-directed research studies [24] (eg, surveys and interviews), and such studies contribute meaningfully to this domain of knowledge. At the same time, such mechanisms rely on researchers’ presuppositions about what questions to ask and what topics are important. In contrast, large-scale analyses of social conversations have the potential to elucidate aspects of drug use about which scholars are unaware, or less aware, but that may be important to facilitating harm reduction. This exploratory work can theoretically be used to identify new research strategies, approaches, and theories around drug use that are grounded in inductive analysis of discourse rather than deduced from existing theoretical frameworks. For example, understanding these informal communities can help public health officials better understand real drug use trends that they might see among younger populations. Similarly, learning about the emotional valence of the discussion of specific substances might help inform context-targeted communication strategies.

On the basis of user demographics, when collecting data in 2022, we perceived that X (Twitter) would be a useful source of observational data to understand how young people feel about several types of drugs as well as about drug use more generally. Until recently, X (Twitter) was the social media platform of choice for researchers in this space due to the large amount of short-form textual data available on the platform through its application programming interface (API) [2,6,11,25]. While recent changes to the API have made research on the platform substantively more prohibitive, recent data sets collected before this change still offer excellent utility. This study used such a data set to better understand the themes, sentiment, and engagement levels for drug-related social media conversations. Principles identified through this work will have utility for natural language processing (NLP) analyses across multiple social media platforms.

### Literature Review

Specific to drug use and outcomes, researchers have used data from X (Twitter) to identify adverse drug reactions using methods from machine learning [26-28], monitor population-level opioid abuse in real time [29-31], study user sentiment about specific types of drugs [32,33], and characterize how young people feel about certain drugs like cannabis and drug use more generally [34-36]. A study by Meng et al [37] using data from X (Twitter) found that the types of drugs people used varied by demographic characteristics as well as geographic characteristics. The authors collected 79.8 million tweets and analyzed 699,757 tweets that were related to drug use to find associations between the sentiment recorded in sentiment-related tweets and zip codes by analyzing which drugs were tweeted about the most often using hashtags. Another study by Stevens et al [36] identified which drugs are discussed by younger populations and identified specific themes indicating how young people discuss drug use on social media. Both studies sampled a large amount of data but qualitatively coded a subset of their data set. Taken together, these studies suggest that using social media as a barometer to understand public sentiment may be a fast way to ascertain public sentiment without having to use advanced surveying methodology while avoiding certain implicit assumptions that might be made in such surveys.

Our research builds on these previous studies using a similar-sized X (Twitter) data set to Meng et al [37]. However, our approach was distinct; we leveraged unsupervised machine learning techniques to computationally identify the main themes in our drug use tweet data set instead of manually analyzing tweets looking for mentions of specific drug names. In our prior analysis of this data set [38], we used latent Dirichlet allocation (LDA) to generate topics based on “street-name” tweets (eg, colloquial terms) or “brand-name” tweets (eg, trademarked or generic terms) [39]. Using that method, we found that tweets that fit into these 2 categories had different themes. Tweets that contained the brand or prescription name of a drug (eg, OxyContin, Vicodin, fentanyl, etc) had a higher likelihood of referring to the impact that drug use has in the context of US politics, political conversations, and society at large. This contrasts with tweets that referred to drugs via their street names (eg, Vikes, Oxys, etc), where individuals would, at times, openly and informally discuss their drug use.

Furthermore, in contrast to tweets using street names, LDA more clearly categorized tweets containing brand names of drugs into specific drug categories, and as noted, many such tweets contained discussion of political events. Tweets containing street names were more difficult to classify using LDA for 2 reasons. First, street names for drugs could often refer to other words with different meanings and contexts, leading to 2 tweets that could contain the same term, for example, “vike,” but refer to different things entirely (eg, Vicodin or the Minnesota Vikings). Second, compared with the brand-name data set, people appeared to use informal terms to discuss drug use in unique or different nonpolitical contexts, leading to more topics being needed to accurately understand the corpus. We concluded from our previous study [38] that unsupervised machine learning techniques could be leveraged to understand how the public perceives drug use on social media but that its utility for categorizing tweets using street names for drugs was lower than for tweets using brand-name drugs.

As noted, our previous work used LDA, which relies on probability distributions and word co-occurrences to determine latent topics. To expand on this work, we leveraged a neural network approach to topic modeling called BERTopic [40]. BERTopic relies on semantic word embeddings instead of word co-occurrence, so the algorithm can create coherent topics by understanding the context of each word from pretrained weights. In addition to using BERTopic to perform topic generation, we conducted sentiment analysis on the data that we had collected to understand the intensity and level of emotions associated with each tweet. As part of a larger discussion on digital surveillance of drug-related communication, we sought to expand our previous work by using a more advanced topic modeling tool, in addition to sentiment analysis, to add further context to the types of drug dialogues that may be occurring on the web and to find whether key differences are observed by the type of drug (ie, brand name vs street name). We used Valence Aware Dictionary and Sentiment Reasoner (VADER) scores [41] to characterize the intensity of emotions of each tweet and determined the mean VADER scores for each topic. BERTopic, a newer sentiment analysis tool, is widely viewed as a more accurate topic generator than LDA. Using these methods, our research was guided by three specific research questions:

1. Using a neural network approach to topic modeling, what key semantic and thematic differences are observed in a corpus of tweets pertaining to a drug’s brand name versus street name?
2. Using a lexicon-based sentiment analysis tool, what lexical differences in sentiment are observed in a corpus of tweets pertaining to a drug’s brand name compared with its street name?
3. Using logistic regression, can we accurately predict the VADER-generated sentiment label of a tweet (ie, positive, negative, or neutral) from a tweet’s engagement metrics?

Findings from this study stand to further refine our data by more clearly identifying content not pertaining to drug use or drug communication. The more refined corpus derived from such an approach, with reduced prevalence of extraneous content, can be further leveraged to construct a drug communication classifier that may better assist in analyzing larger, unstructured language data. Furthermore, by comparing results from LDA, a probabilistic approach to topic modeling, and Bidirectional Encoder Representations from Transformers (BERT), a neural network approach to topic modeling, our study stands to document the growing body of research supporting neural network topic modeling as the optimal choice for unsupervised NLP tasks. Importantly, findings from this study can also inform an additional pipeline to construct a classifier pertaining to drug communication on the web.

## Methods

### Data Collection

Data for this study were collected from X, the social networking website formerly known as Twitter, between October and December 2022 before the discontinuation of its open-access API. To obtain the data relevant to this study, we leveraged the National Institute on Drug Abuse’s list of commonly abused prescription drugs to create the brand-name corpus. See Textbox 1 for a list of all queried drugs, parsed by brand and street names.

**X (formerly Twitter) application programming interface queries by brand name and pseudonym (street name).**

**Brand-name queries (n=31)**

- Xanax, Percocet, Oxycontin, Vicodin, Fentanyl, Opana, Kadian, Avinza, Adderall, Ritalin, Ambien, Sonata, Lunesta, Valium, Librium, Halcion, Ativan, Amytal, Nembutal, Seconal, Roxanol, Duramorph, Actiq, Duragesic, Sublimaze, Tylox, Percodan, Biphetamine, Dexedrine, Concerta, MDMA

**Street-name queries (n=33)**

- Hillbilly Heroin, Oxy, Oxy 80s, Rushbo, Blue Mollies, Black Mollies, Percs, Happy Pills, Barbs, Phennies, Tooies, Downers, Tranks, A-Minus, Zombie Pills, Skippy, The Smart Drug, Vitamin R, Benzos, Benzies, R-Ball, Crystal Meth, Pep Pills, Ludes, Hydros, Idiot Pills, Watson 387, Dexy, Dexies, Ampes, Super Jellies, Speed Pill, Uppers

Using this list as a reference point, we created bots to run strategic queries and Boolean phrases to collect tweets containing a reference to ≥1 prescription or street-drug names. As a comparative study, we triaged all tweets into one of the following two corpora: (1) a brand-name corpus that comprised all tweets with reference to prescription drugs, branded or technical names, and (2) a street-name corpus that comprised all tweets with reference to colloquial names for those drugs. Before cleaning, we had collected 362,216 (38.79%) tweets containing street-name references and 571,564 (61.21%) tweets that contained references to prescription brand-name drugs, totaling 933,780 tweets. After cleaning the data, which involved standardizing the text to identify and remove duplicates, the brand-name corpus contained 245,145 tweets and the street-name corpus contained 170,618 tweets, for a composite sample size of 415,763 (see Parker et al [38] for further insights into the development of this corpus).

### Approaches

#### Overview

In this study, we combined a variety of NLP and machine learning tasks, including those pertaining to theme generation (neural network topic models), dimensionality reduction, and sentiment detection using VADER. We also used an informal qualitative review of our data and exploratory multinomial logistic regression. We explain each briefly below.

#### Neural Network Topic Modeling

Topic modeling refers to an NLP technique that uses a series of calculations to extract latent topics or themes from a collection of related documents or texts. We used a neural network topic modeling pipeline by generating topics using BERT vectors. BERT is a powerful, state-of-the-art transformer-based language retrain model that can understand the context and meaning of words and sentences by comparing input data against a large-scale, pretrained data set. BERTopic is a topic modeling technique that uses BERT vectors to extract latent topics from corpora using one of many pretrained transformer models [42]. BERT’s ability to generate high-quality word embeddings with clustering techniques produces coherent and semantically and contextually meaningful topics from a corpus of documents. Because the meaning of a word can change depending on the context, this is particularly useful for textual data analysis.

#### Dimensionality Reduction

Calculating BERT embeddings generated for corpora is computationally expensive and requires substantial computing power to run effectively. Therefore, dimensionality reduction, the process of transforming high-dimensional data into lower-dimensional data while retaining key elements of the data, is a key component of the topic extraction process. To accomplish this, we used 2 approaches: Uniform Manifold Approximation and Projection, a dimensionality reduction tool that can better detect the complex relationships between tweets on the basis of their language, and k-means clustering (k-means), a popular algorithm used for classification, clustering, and topic modeling, which was used as a clustering algorithm to perform topic modeling on BERT embeddings of the corpus data. The fundamental principle of k-means is to split a data set into k-clusters by defining k-centroid values in feature space. These centroids are initially randomly assigned and used to define the clusters. Through iterative assignment, the centroids are updated on the basis of how the data points are placed in the feature space. The choice of “k,” representing the number of clusters to consider, is a critical parameter that can be tuned to control the algorithm’s sensitivity to local variations in the data.

To find the number of k-topics, we measured the coherence score of different topic configurations. A coherence score [43] is derived from an iterative analysis to identify the optimal number of topics for a given corpus. Coherence scores are a way to evaluate the efficacy of topic models by measuring how well our topics represent the text corpora they are based on. A coherence score ranges from 0 to 1, and larger scores theoretically equate to more interpretable topics.

#### Sentiment Analysis

We used VADER [41] to analyze and score the emotionality of our text. VADER is a rule-based tool for sentiment analysis that uses a specialized lexicon to capture both the polarity (positive, negative, and neutral) and the intensity of the sentiments expressed in a text. Unlike traditional sentiment analysis, VADER focuses on context-dependent emotional tones and accounts for nuanced sentiment expressions. This makes VADER particularly useful in deciphering sentiment in social media text, customer reviews, and informal communication, where conventional sentiment analysis techniques might fall short. VADER uses a lexicon of words and phrases, each of which is assigned a sentiment score based on their emotional connotations. Then, from the word order and sentence structure of a document, the intensity of the sentiment changes. For example, a phrase such as “Yay. Another phone interview” has a different sentiment score from “Yay! Another phone interview!” due to the extra exclamation marks, which would result in an increase in the intensity of the score. Sentiment scores in VADER range from –1 (very high negative valence) to +1 (very high positive valence). The sentiment score associated with a tweet is calculated by adding the individual sentiment valence scores from each word that corresponds to a word in the VADER lexicon and considering the punctuation and capitalization of a tweet to adjust the score accordingly. That value is then normalized from –1 to +1. We refer to this as the normalized, weighted VADER compound score (or compound score more generally). Using this number, we can measure the strength of the emotions associated with a tweet. After finding the sentiment compound score, we then classify the score into 3 labels: positive, negative, or neutral. A *neutral sentiment* is any sentiment where the score is between, but does not include, –0.05 and 0.05 [44]. A *positive sentiment* is defined as any VADER score ≥0.05, while a *negative sentiment* is any score ≤–0.05. We then report the percentage of tweets that are positive, negative, or neutral in our corpus. Given our research questions, we extracted a compound VADER score (with a possible range of –0.99 to 0.99) and a label (positive, negative, or neutral) based on our cutoff criteria. Our use of VADER is strongly supported in computational health science research [45-48].

#### Informal Manual Review

After we extracted latent topics, we applied a sorting function in which tweets in our corpus were assigned to one of the k-corresponding topics on the basis of the presence of topic keywords. Once data in both corpora were sorted into topics, we briefly reviewed a select number of posts for each topic to add context to topic names and keywords. This process is standard for topic modeling analyses, as computers can only extract latent topics and cannot infer deeper meaning with unsupervised NLP methods.

#### Sentiment Label Prediction

All tweets in our study were collected with their engagement metrics, including likes, replies, and retweets. Previous research [49,50] suggests that certain facets of language including affect (or sentiment), tone, and content are associated with highly positive or negative sentiment content, which in turn is associated with higher engagement on social media. While different engagement metrics (likes, retweets, and replies) are associated with different meanings for people [51], individuals engage more with highly inflammatory content [52,53]. However, there is some disagreement about whether positive or negative content is engaged with more frequently [53]. Here, our objective was to determine whether we could predict the sentiment label of a tweet given its BERT-generated topic and the number of likes, retweets, and replies it has. The sentiment label of a tweet is +1, or 0, or –1, signifying a positive, neutral, or negative sentiment polarity for that tweet, respectively. From past research, we know that tweets with highly emotional language are retweeted more and generally receive more engagement [50,52,54]. We hypothesized that knowing the general content of a post (which is what the topic ID will tell us) and how engaged users are with a tweet would allow for accurate prediction of the sentiment label. To test that idea, for each corpus, we created a regression model to find whether labels can be predicted without needing the tweet text. These models contain covariates; engagement metrics (number of likes, retweets, and replies); and generated topic IDs. In addition, we compared this model with another model that used these variables and added the term-frequency–inverse document frequency (TF-IDF) vectorized clean-tweet text as a covariate to understand if word context was needed to accurately predict the sentiment label. TF-IDF vectorization [55,56] is a method to convert the textual information of a document to a numerical representation where each word in the document is converted to a number representing how important that word is in the corpus. This makes it easier to compare how similar 2 documents are in the corpus. In our exploratory regression models, we used the top 5000 features from each corpus based on the generated TF-IDF scores. By comparing these 2 models, we determined the effect that the context of a tweet has on predicting the emotionality associated with the tweet.

To predict the sentiment labels for each tweet, we used a multinomial multivariate logistic regression model. The purpose of this model was to classify tweets into one of the following three categories: positive (+1), negative (–1), or neutral (0) sentiment tweets. We implemented a classifier that used logistic regression to find the label for each tweet. Since we were interested in whether the label itself could be predicted using engagement metrics and the topic ID, we did not use any specific label type as a reference group and used the one-vs-rest heuristic method to classify labels. To evaluate the efficacy of our models, we used the *F*_1_-score, precision, recall, and accuracy metrics to compare all models. The accuracy metric measures how often the predicted label from a model matches the true sentiment label, while the precision metric measures the proportion of true positives found by the model. The recall metric measures the proportion of true positives identified divided by the sum of true positives and false negatives, while the *F*_1_-score can be defined as the harmonic mean of the recall and precision metrics. This score is the definitive measure of how well a model correctly predicts values since, unlike accuracy, it considers how often the model classifies outcomes as false positives and false negatives. We used the macroversion of the *F*_1_-score, recall, and precision metrics to account for label imbalance. These metrics are standard for this type of modeling procedure [57] (for more information on macrologistic regression with *F*_1_-score, recall, and precision metrics, see Tarekegn et al [58] and Manning et al [59]). The *sklearn* package (scikit learn) was used to train and test the regression models, and VADER sentiment analysis tools were used from the VADER sentiment python package [41].

### Ethical Considerations

The study data were collected using the formerly available Twitter API. All study data consisted of public “Tweets” on the Twitter or X platform. For the sake of this study, usernames and location data were not used for any part of the analysis. Collection and analyses of these data was designated by the Indiana University Institutional Review Board as Exempt (#18081).

### Procedure

#### Data Collection

Over 3 months, we continuously collected data via the (formerly) openly accessible X (Twitter) API using the search terms outlined in Textbox 1. For all brand-specific queries (eg, Adderall, Vicodin, Percocet, etc), we created a singular composite data set, hereafter referred to as the brand corpus (n=245,145), after initially collecting 571,564 brand-related tweets. For all colloquial, slang, or other similar mentions of a drug (ie, Addies, Vikes, Perks, etc), we created a second composite data set, hereafter referred to as the street corpus (n=170,618) after initially collecting 362,216 tweets.

#### Data Cleaning for BERT and VADER Tasks

After collecting tweets, we began processing the data ahead of the BERT, VADER, and regression analysis. For each data set, we first created a new column named “clean_text,” where we copied the nonpreprocessed text. From this new column, we then performed our cleaning operations using regular expressions. First, we removed any URLs, the mention symbol (@), emojis, numbers, punctuation, and special characters. Then, we removed any white space present in each tweet to create consistently spaced text. Next, we removed any unnecessary parts of speech using a lemmatizer in addition to stop words, which typically obfuscate the clarity of topic models. For the BERT analysis, we compositely analyzed the text that was entirely preprocessed, in line with standard topic modeling applications. For the VADER analysis, we analyzed the unprocessed text, in accordance with conventional VADER applications, to ensure that the context (including punctuation, adverbs, and adjectives) was considered in the final sentiment score.

#### Coherence Score Calculations

Once the data were preprocessed, we performed iterative topic models with coherence score calculation to identify optimal model fit, beginning with baseline recommendations outlined by Parker et al [38]. To perform an iterative BERT analysis, we tested a range of topic model solutions ranging from 10 to 60 topics, iterating by increments of 10 (eg, *k*=10, 20, 30...60 topics). For the brand-name corpus, we found that a smaller number of topics <10 would be needed to find the optimal coherence score. As such, we tested a range of topics from 5 to 20 in increments of 5 (ie, *k*=5, 10, 15, 20). After each iteration, we calculated a coherence score, which infers the degree to which a human can intuitively understand what a computer-generated topic represents. Higher coherence scores denote greater clarity; lower coherence scores denote lesser clarity. After running all iterations, we identified a different topic solution per corpus. We identified 5 topics (brand-name coherence=0.699) and 40 topics (street-name coherence=0.600) as the optimal topic fit for our data sets. Once we identified the optimal topic solution for the brand and street corpora, we then created a sorting function that triaged all data points into one of the k-respective topics based on keyword matching. After sorting the data, we performed an informal qualitative review to identify the primary topic themes, which were retrospectively named.

#### VADER Analysis

We ran the nonprocessed text through the VADER lexicon. For each entry, we calculated the normalized compound sentiment for each tweet. Then, we labeled tweets as having positive, negative, or neutral sentiments if the compound score for sentiment was ≥0.05, between but not inclusive of 0.05 and –0.05, and ≤0.05, respectively, for each label. This threshold value for sentiment is a common standard when using normalized VADER scores [41]. We reported the mean and SD of the compound sentiment score for both corpora. After labeling tweets as positive, negative, and neutral, we counted the number of tweets that contained each label and compared the percentage of positive, negative, and neutral tweets between corpora.

#### Regression Analysis

For the regression analysis, we used the sentiment labels from our VADER analysis, converting the labels from positive, neutral, and negative to +1, 0, and –1. The data set was split (80:20 ratio) for training and testing, respectively. First, we used logistic regression to predict sentiment labels based on the tweet’s topic ID and specific engagement metrics (ie, likes, replies, or retweets). This was conducted separately for each engagement metric; combining them necessitated establishing a method to appropriately weigh the different engagement metrics, since each engagement behavior implies a different degree of “engagement” (eg, “liking” a tweet takes less effort than writing a reply). Next, we applied a multiclass logistic regression to predict sentiment labels, incorporating the topic ID, engagement metrics, and top 5000 features based on their TF-IDF vectorization. Finally, we applied the Limited-memory Broyden-Fletcher-Goldfarb-Shannon optimizer to optimize the weights in our model. We reported the macroaggregated precision [59], recall, accuracy, and *F*_1_-score metrics among the multinomial models [60,61]. This specific type of aggregation was performed since the distribution of sentiment labels was fairly balanced.

## Results

### Research Question 1: Using a Neural Network Approach to Topic Modeling, What Key Semantic and Thematic Differences Are Observed in a Corpus of Tweets Pertaining to a Drug’s Brand Name Versus a Street Name?

#### Overview

**Figure 1:**
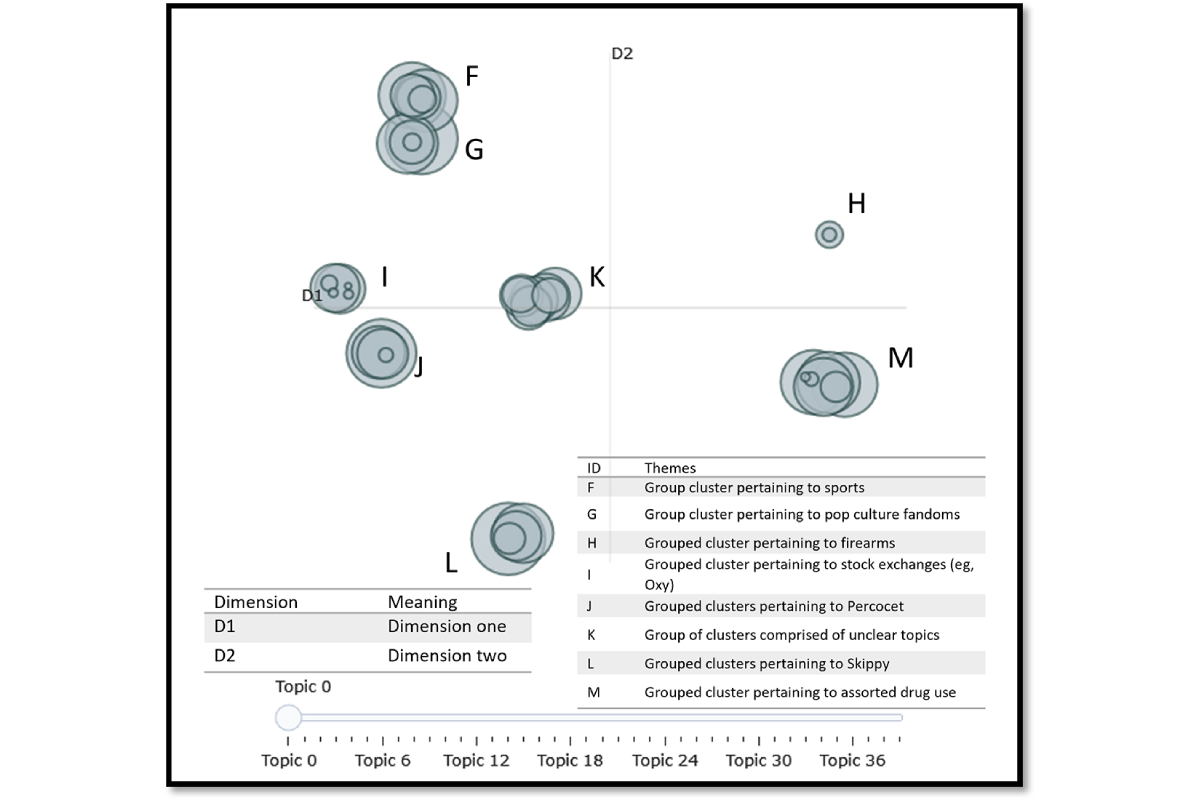
Street corpus intertopic distance map denoting topic overlap. The topics were generated from our BERTopic model, and the themes were decided from qualitative analysis of the posts within each topic. From our iterative BERTopic analysis, the number of topics with the highest coherence of 0.600 was 40.

Our neural network topic modeling pipeline identified several noteworthy differences in the brand and street-name corpora. This includes optimal topic size in either corpus, scope of the topics, and relative clarity in the final models. Table 1 provides information about the 5 topics in the brand-name corpus (the optimal number of topics based on the coherence score measurement). In Table 2, we report on the themes of each cluster as reported by BERTopic. We contrast this with the findings in Table 3, where we searched for 40 topics in the street-name data set. We describe the top 10 words in each topic in Table 3; then, we summarize the meaning of the groups in Table 4. The groups were determined qualitatively in Table 4 by cross-referencing Figure 1, based on which topics were overlapping.

**Table 1:** Brand-name topic ID information, including key terms, count, and percentage of topic ID (n=245,145).

Topic ID	Top 10 search terms per topic	Document count, n (%)
0	Adderall, Ritalin, ADHD^a^, amphetamine, stimulant, medication, prescription, drug, prescribed, meth	76,798 (31.33)
1	fentanyl, cartel, Biden, heroin, illegals, crisis, drug, trafficking, Bidens, epidemic	59,382 (24.22)
2	Psychedelics, LSD^b^, shrooms, psychedelic, drug, ecstasy, weed, pill, cocaine, ketamine	40,001 (16.32)
3	Xanax, anxiety, Vicodin, drug, pill, prescribed, calm, bar, addicted, panic	37,048 (15.11)
4	Sonata, Beethoven, piano, symphony, Mozart, composer, concerto, allegro, Chopin, moonlight	31,916 (13.02)

^a^ADHD: attention-deficit/hyperactivity disorder.

^b^LSD: lysergic acid diethylamide.

**Table 2:** Brand-name group information, including key terms, count, and percentage. The qualitative themes were generated based on the top 10 terms seen in Since only 5 topics were found from the BERTopic model, the topics and groups were able to be matched with each other easily (n=245,145).

Group ID	Overarching themes	Topic IDs in group (topics forming groups in Figure 2)	Document count, n (%)
A	Adderall, Ritalin, ADHD^a^, stimulant use	0	76,798 (31.33)
B	music, concerts, posts unrelated to drug use	4	31,916 (13.02)
C	psychedelics, LSD^b^, hallucinogens	2	40,001 (16.32)
D	Xanax, anxiety, depressants	3	37,048 (15.11)
E	fentanyl, overdose, US politics	1	59,382 (24.22)

^a^ADHD: attention-deficit/hyperactivity disorder.

^b^LSD: lysergic acid diethylamide.

**Table 3:** Individual street topic information, including key terms, count, and percentage (n=170,618).

Topic ID	Top 10 search terms per topic	Document count, n (%)
0	skippy, skippys, skipp, skip, damned, damn, love, darn, f*ck, hell	10,703 (6.27)
1	barb, barbz, barbed, barbarian, beyhive, fav, time, lmao, stardust, bg	10,339 (6.06)
2	percs, perc, perk, perky, leave, n***a, shit, im, bruh, bro	9634 (5.65)
3	playoff, qbs, fumble, nfl, dallas, 49ers, afc, touchdown, offense, qb	8916 (5.23)
4	pill, happiness, smiling, antidepressant, mood, happy, joy, depression, smile, happiest	8480 (4.97)
5	meth, crystal, methamphetamine, crystalmeth, drug, cocaine, heroin, coke, methclouds, addict	8432 (4.94)
6	vikes, vikesbills, losing, winning, playoff, game, win, loss, lose, beat	7986 (4.68)
7	benzodiazepine, benzos, benzo, xanax, antidepressant, prescribing, antipsychotic, medication, ssri, anxiety	7754 (4.54)
8	barb, barbz, nicki, minaj, rapper, rap, lil, nickis, gang, grammy	7313 (4.29)
9	skippy, skippys, taxpayer, tory, cpc, government, trickle, labour, politician, govt	7200 (4.22)
10	cannabis, marijuana, weed, drug, heroin, psychedelics, shrooms, morphine, cocaine, lsd	6926 (4.06)
11	crackheads, perc, lean, crack, crackhead, shrooms, percs, coke, drug, weed	5611 (3.29)
12	upper, lower, higher, high, knockeruppers, taking, pickeruppers, like, hand, took	5401 (3.17)
13	percs, perc, pop, nigga, poppin, bitch, popping, lil, yo, dat	5112 (3)
14	skippy, skippys, fact, pathetic, racist, ignorance, hate, claim, troll, false	5024 (2.94)
15	torch, welder, welding, wgas, oxys, weld, profitable, ox, oxy, kit	4965 (2.91)
16	trading, stockmarket, market, stock, profit, investing, earnings, investment, marketbreadth, sector	4786 (2.81)
17	murdered, victim, peadophiles, murder, 911, twitter, social, room, downer, dont	4690 (2.75)
18	song, release, album, music, 2019, muddy, toe, tpne, weekend, forever	4431 (2.6)
19	janet, dorothy, barb, betty, robert, love, kitty, miss, rachel, dearest	3865 (2.27)
20	yellow, referee, ref, penalty, foul, match, fifa, fifaworldcup, england, worldcup	3813 (2.23)
21	eileen, dexy, dexys, dex, dexies, dexter, dexytools, dexy_buys, dexy_updates, dextools	3805 (2.23)
22	skol, vikes, vikesbites, skolvikes, gopher, game, team, win, hock, winning	3772 (2.21)
23	house, budget, buying, buy, home, fixerupper, fixer, buyer, #shopmycloset, renovation	3592 (2.11)
24	nsfwtwitte, leakedvideos, nsfwtwt, leakedvideo, nsfwtw, nsfwvid, nsfw, discord, skippyleaks, chastitylifestyle	3331 (1.95)
25	grove, downersgrove, hiring, retailjobs, suburb, downtown, downer, st, naperville, chicago	2604 (1.53)
26	debbiedowners, debbie, downer, debby, nancy, gue, karen, dah, boebert, owl	2488 (1.46)
27	peanut, butter, skippy, snack, jelly, jiffy, reeses, chocolate, nuttin, sandwich	1969 (1.15)
28	gain, daily, gme, reduce, wmt, totalday, mixed, sqqq, amp, pt	1874 (1.1)
29	central, basketball, varsity, halftime, chicago, tonight, livestream, tournament, illinois, east	1487 (0.87)
30	56mmuppers, rifle, firearm, ar15, 9mmuppers, 62x39uppers, blackoutuppers, receiver, armed, barrel	1418 (0.83)
31	rushbo, rushbos, rushie, rush, el, miss, limbaugh, limbaughs, bo, linda	619 (0.36)
32	jordanpeterson, peterson, jordan, shooter, manson, follower, serotonin, twitter, walmart, fan	520 (0.3)
33	spy, trader, chatroom, gden, roku, ccl, gmbl, rgr, rcl, wfc	410 (0.24)
34	pigeon, meth, prison, detained, correctional, backpack, carrying, caught, arrested, smuggle	390 (0.23)
35	blackoutuppers, grape, blackout, upper, receiver, stainless, 316, 300, defense, tactical	377 (0.22)
36	volume, callput, xle, plug, overview, 192, ratio, energy, xrxoxy101, total	167 (0.1)
37	oxy_usdt, wrx_usdt, oxy_usdtsuggested, aln_usdt, xyo_usdt, wncg_usdt, xprt_usdt, usdt, lamb_usdt, aioz_usdt	166 (0.1)
38	meth, jordanpeterson, peterson, serotonin, manson, stimulant, shooter, follower, jordan, cybermen	165 (0.1)
39	stock, group, chatroom, trade, trxc, amd, astx, mgm, gmbl, amzn	83 (0.05)

**Table 4:** Street name grouped topics including overarching themes, document count, and percentage. The qualitative themes were generated based on the top 10 terms seen in From the 40 topics, 8 groups were found from the overlapping topics seen in Figure 1. Each of the 8 groups has a unique theme associated with it, with differing numbers of topic per theme (n=170,618).

Group ID	Overarching themes	Topic IDs in group (topics that form groups in Figure 1)	Document count, n (%)
F	Group cluster pertaining to sports related topics and themes	3, 6, 20, 22, 29	25,974 (15.22)
G	Group cluster pertaining to pop culture fandoms (eg, the Barbz, a Nicki Minaj fanbase)	1, 8, 19, 31	22,136 (12.97)
H	Grouped cluster pertaining to firearm dialogue and online sales	30, 35	1795 (1.05)
I	Grouped cluster pertaining to stock exchanges (eg, Oxy)	15, 16, 32, 36, 37, 39	10,687 (6.26)
J	Grouped clusters pertaining to Percocet use and access	2, 11, 13, 33	20,767 (12.12)
K	Group of clusters comprising unclear, uncertain topics	12, 17, 18, 21, 23, 24, 25, 26	30,342 (17.78)
L	Grouped clusters pertaining to “Skippy” as a peanut butter brand, drug, and political figure	0, 9, 14, 27	24,896 (14.59)
M	Grouped cluster pertaining to assorted drug use, including meth, crack-cocaine, and others.	4, 5, 7, 10, 28, 34, 38	34,021 (19.94)

#### Brand Corpus

**Figure 2:**
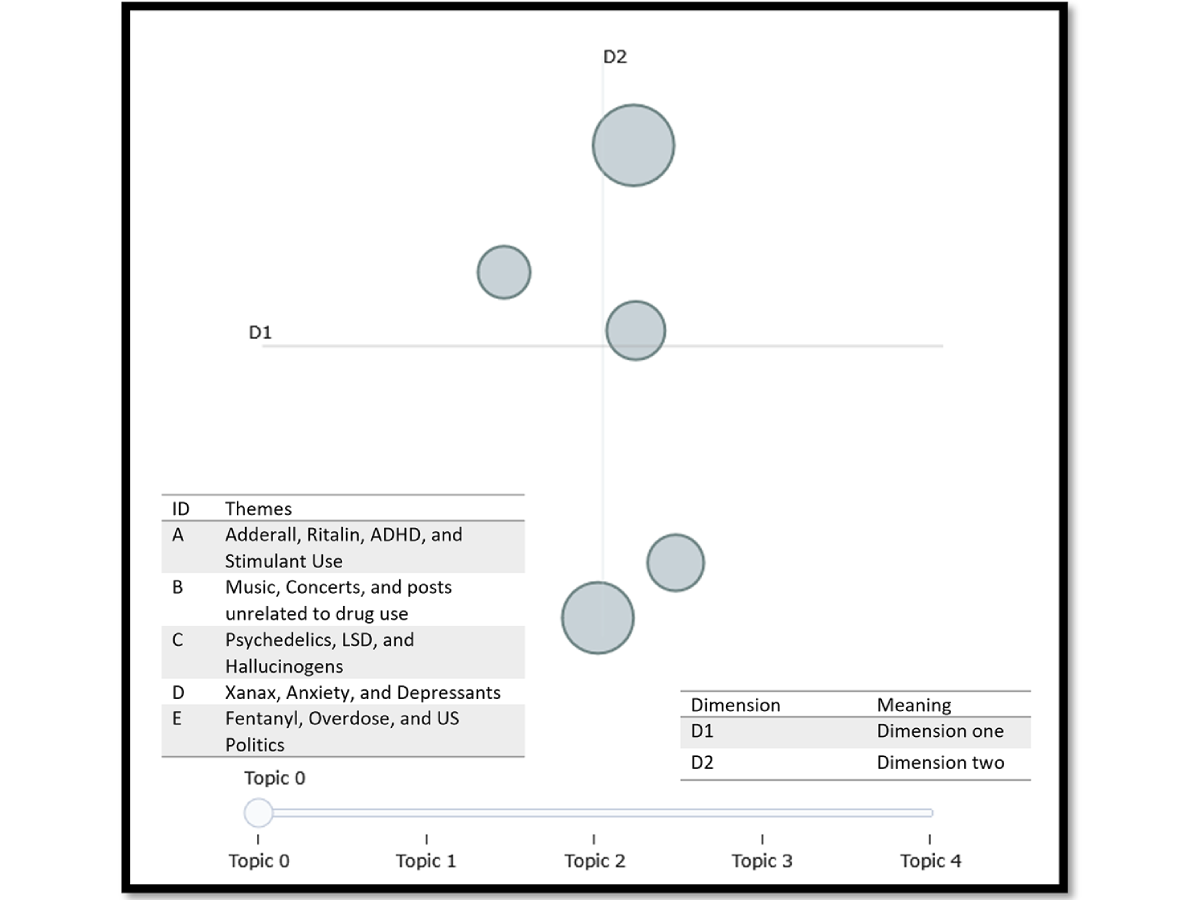
Brand corpus intertopic distance map denoting topic overlap. The topics were generated from our BERTopic model, and the themes were decided from the qualitative analysis of the posts within each topic. From our iterative BERTopic analysis, the number of topics with the highest coherence of 0.699 was 5. ADHD: attention-deficit/hyperactivity disorder; LSD: lysergic acid diethylamide.

Our iterative BERTopic analysis yielded a 5-topic solution (coherence=0.699). Figure 2 provides a visualization of our data using an intertopic distance map. This map allows us to infer the relative similarity (or high correlation) and dissimilarity (or low correlation) of each topic relative to one another. From Figure 2, we can infer 5 mutually distinct topics, which is evidenced by the absence of overlap between clusters. When reviewing each cluster’s keywords, we further inferred that each topic pertained to an overarching drug class. Group A principally referred to stimulant use; group B referred to music, concerts, or tweets otherwise not pertaining to drug use; group C referred to psychedelics and hallucinogens; group D referred to depressants; and group E referred to fentanyl use and overdose.

Table 1 offers further context regarding the distribution of topics, while Table 2 shows the relevant groupings and themes based on the topics in Table 1 and the clustering shown in Figure 2. We note that the group ID in Table 1 corresponds to the clusters labeled in Figure 2. The 2 groups with the greatest prominence were group A (76,798/245,145, 31.33%; *Adderall*, *Ritalin*, *ADHD*, and *stimulant use*) and group E (59,382/245,145, 24.22%; *fentanyl*, *overdose*, *US politics*), comprising >55% of the brand-name corpus. Regarding stimulant use, or group A, we observed a variety of different subthemes, including recreational use (tweet: “being on Adderall is so fun bc i just spent 30 minutes watching tik toks of snoopy dancing to different songs”) and as a current events topic (tweet: “@JoeBiden what is your plan to fix the adderall shortage?”). The second most prominent theme, fentanyl, or group E, was largely centered on discussing the drug in a strongly political and current events context, often spanning overdose rates and the impact of immigration on fentanyl availability (tweet: “They were killed by people with guns. BTW, you also forgot 108,000 people killed by open borders fentanyl in the last year*.*”). Notably, we did not observe much discussion about the recreational use of fentanyl in our data. Groups C (psychedelics) and D (depressants) largely covered recreational uses of these drugs. However, we did observe a body of tweets advertising the sale of hallucinogenic products in states where their use is ostensibly legal (tweet: “I love microdosing and I gladly recommend [redacted] on Instagram they got shrooms LSD dmt MDMA fast shipping and delivery*”*). We classified 13.02% (31,916/245,145) of our data into group B, which we qualitatively deemed to contain posts not specific to drug use. Recurring mentions in group B included music, concerts, and car brands (tweet: “The suspect fled the scene in a white, four-door, Hyundai Sonata with an obscured North Carolina temporary tag, according to police”; tweet: “Nice piece, devils trill sonata is a good choice 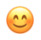.”), which may be explained by the name, Sonata, and its various associations.

#### Street Corpus

Our iterative BERTopic analysis yielded a 40-topic solution for the street corpus (coherence=0.600). Figure 1 visualizes our topics using an intertopic distance map where the overlap denotes high topic correlation, and sparsity indicates low topic correlation. Unlike the brand corpus, which contained 5 nonoverlapping topics that could be easily generalized into specific themes, the 40 topics associated with the street corpus had various degrees of overlap, which indicates highly similar, or correlated, topics. When reviewing the keywords associated with each of the 40 topics and associated distributions (Table 3), we categorized our data further along 8 overarching themes as further illustrated in Figure 1. More specifically, clusters associated with group F were largely about sports, group G about pop culture fandoms, group H about firearms, group I about the stock exchange, and group J about Percocet, while group K contained unclear focus, group L contained a variety of tweets about “skippy” in various contexts, and group M contained posts about assorted drug use.

Table 4 offers further context regarding general topic distribution and group clustering. There were fewer topics pertaining exclusively to drugs and drug use in the street corpus. In place of such drug-related conversations, we instead observed a disjointed collection of topics that were either not clear (group K: 30,342/170,618, 17.78% representation) or more succinctly focused on non–drug-related topics including sports (group F: 25,974/170,618, 15.22% representation), pop culture fanbases (group G: 22,136/170,618, 12.97% representation), firearm dialogues and sales (group H: 1795/170,618, 1.05%), stock prices and sales (eg, OXY; group I: 10,687/170,618, 6.26% representation), and myriad uses for the term “skippy,” (group L: 24,896/170,618, 14.59% representation). Importantly, these non–drug-related topics all contained the appropriate query name, yet the foci of the tweets were decisively not drug related. For example, tweets regarding sports referenced the Minnesota Vikings using their common nickname, “the vikes” (tweet: “Ya, the unknown clock. The vikes would get screwed on that one. I promise you that”). For fandom, we observed a substantive body of tweets about Nicki Minaj’s fanbase, commonly referred to as “the barbz” (tweet: “Barbs weird always wanting Nicki to be friends with people who don’t like her”). Barbs, or barbz, also refers to a common street name for barbiturates. For stock prices, tweets referenced Occidental Petroleum Corporation, listed on the US Stock Exchange, as “OXY” (tweet: “I’m also very bullish on $OXY stock”). Skippy often referenced a peanut butter brand (tweet: “id honestly put skippy peanut butter in my top five favorite foods”) and also referenced Canadian politician Pierre Poilievre, leader of the Conservative Party of Canada [62] (tweet: “Yet another one that Skippy, nor the Conservatives have a solution to address. Just like when they voted against dental care for children.”). However, despite the noise inherent to these conflated topics, we also observed numerous instances in which a tweet referenced a particular query and was, in fact, drug related.

After an informal qualitative review, we determined that approximately 32% of posts (groups J and M) pertained directly to drug use. In contexts where a post was about a specific kind of drug use, we observed more direct statements about recreational use. We also determined groups J and M largely, and nearly exclusively, referred to drug use in a recreational and often light-hearted context (tweet: “Honestly, most of the prosecutors I know were also coked out—it’s refreshing to see a cop who loves downers so much”; tweet: “Ohh yeah ladies, I forgot to mention they had me on downers and I smoked pot*.*”).

#### Contrast Between Corpora

We observed both obvious and nuanced differences between corpora. First, the BERT-identified optimal number of topics differed between the brand corpus and the street corpus, which may reflect the relative consistency of brand-related content and the broad diversity of the street-related content. Indeed, in the brand corpus, we observed consistent discussions of a drug in a recreational context. However, we also consistently observed how certain drugs, including fentanyl and Adderall, we often discussed in a current events context (ie, the nationwide Adderall shortage) or in a sociopolitical context (ie, immigration and its effects on fentanyl distribution along the southern border). These more formal pockets of conversation were almost entirely lacking in the street corpus where only a small portion of the tweets explicitly mentioned drug use; nevertheless, we acknowledge that a full review of each tweet was not undertaken. When it was apparent that a tweet contained an appropriate query but no mention of a drug, we observed the content pertaining to the term’s other potential applications or uses. Unique to the street corpus seemed to be more positive mentions of a given drug, typically in a recreational use context or as a light-hearted exchange. Many tweets in the street corpus also had limited context, making it difficult for a computer or members of the study team to appropriately categorize (tweet: “OMG. I love the barbz so much”; tweet: “Gotta love my Vikes”). Thus, despite leveraging a more refined algorithm to conduct a topic modeling analysis (in contrast to our prior use of LDA), there was still an inherent messiness to these data that require further refinement and consideration.

### Research Question 2: Using a Lexicon-Based Sentiment Analysis Tool, What Lexical Differences in Sentiment Are Observed in a Corpus of Tweets Pertaining to a Drug’s Brand Name Compared With its Street Name?

In addition to content differences in the brand and street-name BERTopic analysis, we also identified affective similarities and differences using VADER, a lexicon-based sentiment analysis tool. For this analysis, we extracted the compound VADER score per tweet, which ranged from –0.99 to 0.99, and emphasized a tweet’s valence intensity. We also extracted the sentiment label (positive, negative, or neutral) based on our cutoff criteria. We observed key differences by score and label. First, the mean VADER compound score for the brand corpus was between –0.05 and 0.05, showing that the mean sentiment was neutral, while the mean compound score for the street corpus was >0.05, indicating an inclination toward positive sentiment in the street corpus (mean brand compound score –0.0082 SD 0.477; mean street compound score 0.11 SD 0.478). However, the SD for the compound scores was large in both corpora (0.47), and this suggests that we cannot broadly generalize the sentiment in the street or brand corpus as being predominantly positive, negative, or neutral.

Table 5 shows the percentage of tweets in each corpus that fit within a specific sentiment label. These percentages do not account for the magnitude of a tweet’s sentiment score. Tweets that were only slightly positive (eg, 0.051) were labeled as having positive sentiment and were categorized alongside extremely positive tweets (eg, 0.80). In other words, our findings indicate both the overall magnitude of sentiment across all tweets in a corpus (the aforementioned mean compound scores) as well as the prevalence of tweets classified with each sentiment label according to our established cutoff scores (Table 5).

One explanation for lower average VADER scores in the brand corpus may be the political nature of a substantive body of these tweets. For example, tweets about fentanyl often emphasized overdose, border security, and other similarly tense political dynamics, which were largely absent from the street corpus. This distinction may also explain the greater presence of tweets tagged with a “positive” VADER value in the street corpus (77,543/170,618, 45.45%) versus the brand corpus (88,826/245,145, 36.23%). Other insights gleaned from VADER include a smaller number of tweets tagged as negative in the street corpus compared with the brand corpus (street: 47,603/170,618, 27.9%; brand: 86,586/245,145, 35.32%). Both corpora contained similar amounts of posts with a neutral sentiment.

**Table 5:** Sentiment percentages for brand and street corpus on the basis of computer-assigned sentiment labels (positive, negative, or neutral).

Corpus	Positive sentiment total	Negative sentiment total	Neutral sentiment total
Brand name	36.2	35.3	28.5
Street name	45.4	27.9	26.7

### Research Question 3: Can We Accurately Predict the VADER-Generated Sentiment Label of a Tweet (ie, Positive, Negative, Neutral) From a Tweet’s Engagement Metric?

When performing logistic regression with the brand-name corpus, the model excluding the text of a tweet as part of the features was 38.5% accurate on average across all engagement metrics compared with the model including tweet text as a feature where the model was 82.8% accurate on average. Similarly, the average model accuracy in the street-name corpus for the model excluding tweet text was 46.7% accurate, while the model including tweet text was 85.4% accurate. In both corpora, the models that included the text of a tweet as a feature when performing TF-IDF vectorization were more accurate by around 40%. We noted negligible differences in accuracy when comparing the likes, retweets, and replies models to each other within each corpus. The macro *F*_1_-scores were even more different between the models that did not use the vectorized text (brand=0.231; street=0.214) compared with the models that did use the vectorized text (brand=0.828; street=0.854). Summary statistics are shown in Table 6.

**Table 6:** Summary statistics for regression models^a^.

Corpus			Accuracy (%)		Precision (%)		Recall (%)		*F*_1_-score (%)
**Brand name**									
	Without text	38.5		58.3		33.5		23.1	
	With text	82.8		82.8		83		82.8	
**Street name**									
	Without text	46.7		35		33.4		21.4	
	With text	85.4		84.8		84.6		84.7	

^a^For both the street- and brand-name corpus, we find that adding the text of the tweet as a feature to our regression model greatly improved the accuracy, precision, recall, and *F*_1_-score compared with the model that did not incorporate this feature.

## Discussion

### Principal Findings

#### Overview

This study used a neural network approach to topic modeling (BERTopic) to examine 2 contemporaneous corpora of tweets selected for brand and street-name drug references. Interestingly, differences in the interpretability between the corpora that we first observed with LDA [38] remained salient with this more advanced approach. Then, using VADER, we identified that the street-name corpus has a larger inclination toward positive sentiment, while the brand-name corpus contains similar amounts of tweets labeled positive and negative. Finally, we combined the results from the topic model and sentiment analysis to create predictive models (logistic regression) to estimate sentiment labels from the topic ID and engagement metrics and compared the accuracy of the models that included the vectorized tweet text as a covariate and the models that did not.

#### Topic Analysis

BERTopic, in combination with Uniform Manifold Approximation and Projection and k-means clustering, yielded statistically coherent clustering of topics, although the outputs for the street-name corpus were more difficult to interpret and generalize. The tweets in the brand-name corpus discussed different drugs in the context of their intended uses, as well as how certain drugs were perceived to relate to ongoing political or social issues. The brand-name data set could be reduced to 5 major themes: broad discussion about fentanyl use and its discussion in a sociopolitical context; stimulant use (eg, Adderall, Ritalin, etc); discussion about music and car models related to the word “sonata”; psychedelic use; and discussion about anxiety-related medication (Xanax). The discourse about fentanyl was especially varied, with many topics containing posts relating to politics, immigration, border security, and, in some cases, actual use. This differed from how people discussed Adderall; in our data, people were concerned about the 2022 Adderall shortage [63] and were interested in how to use the drug safely. As we indicated in the *Results* section, Sonata, the brand name of a sleep aid, tended to capture tweets about music and the Hyundai Sonata car model, and those tweets formed the only topic and category that was not drug related.

For the street-name corpus, the BERTopic model with the highest statistical coherence score produced 40 topics, many of which overlapped and were not necessarily related to drug use. Only 32.11% (54,788/170,618) of all tweets were sorted into topics that pertained primarily to drug use, allowing the inference that most posts pertained to nongermane topics. Observationally, this was because many street names for drugs can refer to a variety of real-world concepts or phenomena (eg, words do not necessarily refer to a drug without additional context). Previous research supports the idea that machine-based NLP approaches may struggle to parse content containing street names for drugs effectively [38,39,64]. In the street-name corpus, 6 of the 8 clusters were sorted around terms unrelated to drug use. Out of these 6 clusters for the street-name corpus, 4 (67%) clusters (60,592/170,618, 35.51% of all posts) contained themes relating to football, fandoms, firearms, and the stock market. The last 2 clusters were even more difficult to categorize: we could only find general themes relating to the word “Skippy” (sometimes used colloquially to refer to stimulants) for one, and the other did not appear to us (as human interpreters) to have a core theme, although the NLP approach had a computational reason for generating the topics and cluster.

Comparing the topics in the 2 corpora, 10 (25%) out of 40 topics in the street-name corpus contained <1% of all posts, whereas the brand-name corpus had only 5 topics total. The street-name corpus contained many niche discussion topics compared with the few general themes of the brand-name corpus. On the basis of our findings from the BERTopic output, we suspect that refining a complex data set of this size by eliminating content that is not drug specific would be arduous. However, in moving from LDA [38] to BERTopic (a more refined algorithm), we were better able to identify pockets of conversation that were not drug specific and were better able to tag them appropriately. Future research should consider additional work in data refining and classifier building with the street-specific data set.

#### Sentiment Analysis and Predictive Modeling

We used VADER to assess the sentiments of tweets and found that both corpora contained tweets with a wide range of sentiments. Interestingly, we found that the street-name corpus had a larger proportion of positively labeled tweets compared with the brand-name corpus. In our study, the terminology categorization for street-drug terms was complex, which may raise questions as to VADER’s applicability. However, VADER’s original validation study was particularly successful at classifying tweets or microblog text (vs other forms of text), outperforming even human raters, and the dictionary of lexical features was designed, in principle, to be domain agnostic [41]. This increases our confidence in the VADER-based assessment of the data. We hypothesize that the street-name corpus was made up of many topics that are unrelated to drug use. Therefore, we suspect that many positive tweets were support from fans, such as fans of Nicki Minaj (barbs) and the Minnesota Vikings (Vikes). However, this analysis pipeline was not able to directly link words and sentiment, so we cannot be sure whether that was the case.

Since the language features associated with emotionality were based on the VADER lexicon, we can know *what* kinds of things were scored as positive but not *why* those features were used to express a certain sentiment. Understanding the motivations behind positive communication is an important next step in understanding how individuals feel about drug use at scale. Arguendo, it might be the case that lexical features (eg, words, capitalization, context, punctuation, etc) associated with positive sentiment occur more often in drug discourse during events (eg, concerts) than drug discourse referring to isolated or solo use. To truly understand why individuals feel a certain way about different types of drug use would require additional deep qualitative methods and analysis. We used multinomial logistic regression to understand if we could predict the sentiment label or emotionality of a tweet using information about the tweet’s topic and how engaged users are with the tweet. We tested permutations of regression models that either (1) included tweet text as a covariate or (2) did not include it. We found that the models including the tweet text as a covariate explained more variation in tweet sentiment (by approximately 60% according to the macro *F*_1_-score) than the models that did not incorporate text as a feature. This result was consistent across both corpora, showing that the generated topic ID and engagement metrics were not sufficient to predict the sentiment of a given tweet. Given the variables to which we had access, the only way to accurately predict tweet sentiment was to use the language itself. This means that aspects *about* a tweet, such as what it discusses (its topic ID) and how engaged people are with a tweet (number of likes, replies, and retweets), cannot be used to accurately predict the emotionality of a given tweet. This speaks to the diversity of opinions within a topic and how difficult it is to understand the sentiment of a tweet without knowing the full context within a post. Without the full context, we cannot predict whether a tweet about drug use will have positive or negative sentiment, even if we know what drug is being discussed and how well engaged people are with a post.

#### Comparison to Previous Literature

In the peer review for a paper on our previous LDA model (Parker et al [38]), reviewers suggested that an appropriate next step would be the use of neural network modeling, which we performed here. The results of the BERTopic model support the conclusions from the LDA model. Specifically, the brand-name corpus was more easily categorized by a machine-based approach than the street-name corpus. As before, this difference seemed attributable to the fact that many of the words in the street corpus do not have a clear meaning outside of a narrow context. For instance, the word “Skippy” can refer to methylphenidate (eg, Ritalin), a brand of peanut butter, or a Canadian politician. In contrast, “fentanyl” has an unambiguous meaning even without context.

The most obvious difference between the models is the number of topics generated. In our prior work, the LDA model generated 20 topics for the brand-name data set, while in this paper, the optimal BERTopic model was able to use 5 topics to cluster all posts. In contrast, the harder-to-parse street-name corpus resulted in more similar numbers of topics for LDA and BERTopic (35 and 40, respectively). The BERTopic analysis could more clearly delineate the different topics of discussion based on word context, allowing for an increased number of topics for the street-name data set and fewer topics in the brand-name corpus since discussion in the brand-name corpus is more homogenous and easily categorizable. The BERTopic model generated more cohesive themes than the LDA model due to pretrained BERT embeddings, which accurately captured the semantic relationships between words; thus, words with multiple meanings are better understood and categorized. In contrast, LDA uses word co-occurrence to generate topics for tweets, so LDA topic models might group documents together into the same topic that have the same word although this word is used in different contexts. As an example, “Adderall” can co-occur alongside other words like “anxiety” and “Ritalin.” In Parker et al [38], the LDA model created 4 separate topics relating to Adderall use and 1 topic relating to the Adderall shortage. However, as we see from our BERTopic model, the more sophisticated algorithm was able to condense those same 4 topics into 1 topic relating to Adderall use, while discussion about the shortage was grouped into the topic relating to the intersection between politics and drug use.

Previous work by Nasralah et al [65] used LDA to better understand the most-discussed topics relating to the opioid epidemic by analyzing 503,830 tweets and filtering tweets via an evaluation matrix. Similar work [66,67] analyzing people’s reactions to the opioid epidemic has been conducted using textual analysis algorithms to find themes in X (Twitter) data. A study by Tassone et al [68] used convolutional neural networks and other deep learning techniques to classify whether tweets about drug use were encouraging drug use (positive) or discouraging drug use (negative) and created synthetic tweets on drug use based on real tweets about drugs. While that approach also incorporated sentiment, our definition of a positive or negative tweet was dependent on the VADER classification instead of defining based on whether a tweet encourages or discourages drug use. In addition, we used a semisupervised technique (BERTopic) to classify tweets into general themes. Many studies [32,69,70] that identify themes for a collection of tweets pertaining to drug use using manual annotation methods, including inductive and deductive qualitative coding, have also been conducted. In 2022, Al-Garadi et al [71] used LDA and VADER scores to understand the different reasons for nonmedical prescription drug use. Cavazos-Rehg et al [34,35] focused on a single drug, marijuana, and how young people discuss marijuana use and react to popular accounts that discuss marijuana use. Both studies from Cavazos-Rehg et al [34,35] assessed sentiment using Twitter, but instead of analyzing sentiment using VADER, they used a crowd-sourcing service to code the sentiment of the tweets. In contrast, we used a classifier model across a wide variety of prescription drug conversations on Twitter rather than using human coders.

#### Strengths and Limitations

Our study’s strength lies in the cohesive topics generated by BERTopic, which enabled a clear understanding of the general themes of discussion in the street- and brand-name corpora. However, there are some limitations to our study. First, we cannot distinguish *why* the amount of positive sentiment differed between the brand and street tweets. The VADER analysis that we performed was descriptive in nature, and although we found the sentiment label and compound score of each tweet, we could not summarize why X (Twitter) users expressed positive or negative sentiments about a drug. Some form of stance detection would have to be conducted to better understand how different users feel about specific drugs. From the VADER scores, we can only identify aggregate trends regarding sentiment and not make conclusions about how individuals feel about specific types of drugs.

In our text-comprehensive regression models, we classified the sentiment labels of tweets with a macro *F*_1_-score of 82.8% in the brand-name corpus and 84.7% in the street-name corpus. Our modeling shows that sentiment labels can best be predicted using the cleaned text of a tweet as part of the feature set including engagement metrics and topic ID. However, without the text of a post, the *F*_1_-score fell to 23.1% in the brand-name corpus and to 21.4% in the street-name corpus. This points to a limitation of topic modeling, that it is primarily an exploratory form of analysis that cannot tell us about the emotionality of a data set. Topic models can help researchers find the general ways how people are discussing a topic, but these topics can neither be used to predict the sentiment within the topic, nor, more obviously, allow deeper inferences about motivations and intentions.

We were also limited by VADER, which is a lexicon-based sentiment analysis tool. Although the use of VADER is widely supported in the literature, there are concerns that VADER scores could be biased due to the overrepresentation or absence of certain words in the lexicon. In our case, certain slang terms for prescription drugs such as “perc” or “fent” are not present in the VADER lexicon as well as certain prescription drug names like Adderall or Ritalin. For our work, we were more interested in the *context* around certain prescription drug names and slang terms. We wanted to understand the emotional affect around certain terms, not necessarily the affect of the term itself. For future work, more work could be done to expand the VADER lexicon to include slang terms in addition to prescription drug names.

One final limitation is the lack of generalizability in our study. From the time we collected our data, Twitter has been rebranded to X, and the number of active users, the way that users interact with the site, and the algorithm to show users’ content have all changed. We are not able to replicate our study since acquiring the volume of data that was available in the past is not feasible. The “infoveillance” component of our analysis is also put under question since geotagging is no longer available. The future of this type of research must be found on other social networking platforms, such as Facebook, Instagram, and BlueSky, which offer first-party APIs to track their data, and through platforms like PushShift, which is a third-party API for Reddit data.

#### Implications

Our findings broadly illustrate the importance of using more advanced computational approaches to mine social media data for conversations mentioning prescription drugs. In this section, we offer some practical implications of our study, including the importance of a refined data set for classifier construction and the need for more advanced sentiment analysis tools.

Our BERTopic model classified the street- and drug-name corpora into a coherent set of individual topics, leading to a higher number of topic clusters in the street-name data set and fewer (only 5) topics for the brand-name data set. By leveraging BERTopic and regression models, we were able to further refine our data set, capturing more nuanced topic meaning to create a future classifier pertaining to web-based communication about drug use. More importantly, we were able to further isolate extraneous content (ie, tweets about cars, fanbases, and sports teams), which, theoretically, would impede the ability to train an accurate classifier. We have taken the first steps to build this classifier by identifying extraneous content. The next step would be to begin a manual annotation process of the refined data set using qualitative expertise to “tag” our data and begin a test-retest approach with training and validation data.

Using VADER, we identified tweets as having positive, negative, or neutral sentiments. Then, we compared the percentages of positive, negative, and neutral tweets between the 2 corpora. This type of analysis allows us to characterize the sentiment in aggregate for the brand and street corpora. To further understand the sentiment that users on X (Twitter) have toward certain drugs, we need to perform more text filtering to find what specific words and phrases are used with certain drug-related words. The next steps include conducting an analysis to identify the lexicon surrounding the street and brand names of prescription drugs to form a better understanding of how certain drugs are discussed. With a more refined data set enhanced by qualitative coding, we may begin to build a training data set that could contain social media illicit drug use conversation data useful for designing health communication interventions.

### Conclusions

This work has shown how data from X (Twitter) can be used to identify topical trends surrounding both informal and formal discussions of drug use among users on the platform. Our work combines topic modeling and sentiment analysis to give greater detail on how users on X (Twitter) feel about different types of prescription drugs. Consistent with Parker et al [38], we found that colloquialisms used in the street-name corpus disguise how people discuss drug use. The improved clustering offered by BERTopic allowed us to identify cohesive themes in the street- and brand-name corpora. The clear themes shown in the brand-name corpus contrast with the difficulties in parsing how individuals discuss street-name prescription drug use. From our literature review, we could not find many other works that captured the difficulties in trying to understand how individuals discuss street-name drug use. This points to a potential gap in the drug-discussion literature on how to analyze drugs when their street names are used. Furthermore, VADER analysis detected more positive sentiment among discussions in the street-name corpus compared with the brand-name corpus. Regression analysis of this classifier model determined that predicting the sentiment of drug use discussion is difficult without the full discussion context; topic and engagement metrics alone were insufficient to predict the sentiment of a street- or brand-name tweet.

## Abbreviations

API: application programming interface
BERT: Bidirectional Encoder Representations from Transformers
LDA: latent Dirichlet allocation
NLP: natural language processing
TF-IDF: term-frequency–inverse document frequency
VADER: Valence Aware Dictionary and Sentiment Reasoner

## Data Availability

The data sets generated during and analyzed during this study are available from the corresponding author on reasonable request.
